# AgentMol: Multi-Model AI System for Automatic Drug-Target Identification and Molecule Development

**DOI:** 10.3390/mps8060143

**Published:** 2025-12-01

**Authors:** Piotr Karabowicz, Radosław Charkiewicz, Alicja Charkiewicz, Anetta Sulewska, Jacek Nikliński

**Affiliations:** 1 Department of Clinical Molecular Biology, Medical University of Bialystok, 15-269 Bialystok, Poland; radoslaw.charkiewicz@umb.edu.pl (R.C.);; 2 Center of Experimental Medicine, Medical University of Bialystok, 15-369 Bialystok, Poland; 3 Department of Analysis and Bioanalysis of Medicines, Medical University of Bialystok, 15-089 Bialystok, Poland

**Keywords:** drug discovery, chemical language model, GPT-2, convolutional neural networks, LangGraph agent

## Abstract

Drug discovery remains a time-consuming and costly process, necessitating innovative computational approaches to accelerate early stage target identification and compound development. We introduce AgentMol, a modular multimodel AI system that integrates large language models, chemical language modeling, and deep learning–based affinity prediction to automate the discovery pipeline. AgentMol begins with disease-related queries processed through a Retrieval-Augmented Generation system using the Large Language Model to identify protein targets. Protein sequences are then used to condition a GPT-2–based chemical language model, which generates corresponding small-molecule candidates in SMILES format. Finally, a regression convolutional neural network (RCNN) predicts the drug-target interaction by estimating binding affinities (pKi). Models were trained and validated on 470,560 ligand–protein pairs from the BindingDB database. The chemical language model achieved high validity (1.00), uniqueness (0.96), and diversity (0.89), whereas the RCNN model demonstrated robust predictive performance with R^2^ > 0.6 and Pearson’s R > 0.8. By leveraging LangGraph for orchestration, AgentMol delivers a scalable, interpretable pipeline, effectively enabling the end-to-end generation and evaluation of drug candidates conditioned on protein targets. This system represents a significant step toward practical AI-driven molecular discovery with accessible computational demands.

## 1. Introduction

A molecule takes approximately 13.5 years to reach its point of approval, with total research and development costs estimated at roughly $2.6 billion [1]. Identifying drug targets, new molecules, and their interactions is pivotal for drug discovery. Drug developers seek novel ways to invent new drug compounds and characterize their drug target interactions (DTI) more effectively and efficiently [2]. As biological data becomes increasingly complex and diverse biomedical information sources continue to expand, there is an urgent demand for innovative computational approaches capable of integrating and analyzing these extensive datasets. Artificial intelligence-driven methods can enhance the speed and accuracy of these tasks by leveraging vast datasets, enabling researchers to explore new possibilities in molecular design and interactions, ultimately improving the efficiency of drug discovery pipelines [3].

The first step in drug development is to identify disease target. Artificial intelligence (AI) tools, especially large language models (LLMs), for automating literature reviews and data extraction have become an integral part of contemporary drug discovery [4].

Searching for the molecule and its DTI is a second, significant component of the drug discovery process. Based on the functionalization approach, computational DTI determination can be categorized into ligand-based methods, which use only the compound features for modeling, and structure-based techniques, which utilize the structural features of both compounds and target proteins [5]. DTI prediction methods can also be classified into two main categories based on their task. The first is the binary classification approach, where prediction is traditionally performed as a binary process, producing an output of either active (i.e., binder) or inactive (i.e., non-binder) [6]. However, binary classification does not capture how strongly drugs bind to proteins, which can influence their effectiveness and limit their utility in virtual screening. Therefore, recent studies have focused on predicting drug-target binding affinity by constructing regression models instead of classification models [7]. Regression models reflect binding strength by predicting affinity values using measurements such as the inhibition constant (Ki), dissociation constant (Kd), or half-maximal inhibitory concentration [7]. The dissociation constant quantifies the affinity between a protein and its ligand, whereas, for enzymes and their inhibitors, Ki is equivalent to Kd. Deep learning, a subset of machine learning, has achieved remarkable success in DTI predictions. For instance, Ozturk et al. developed a deep learning–based model that relied solely on drug–target sequence information. This research introduced a novel deep learning model based on convolutional neural networks (CNNs) for drug–target affinity prediction, utilizing protein and 1D drug character representations [8]. Several CNN-based models have been developed as of 2025 [9,10,11].

In recent years, generative AI research has increasingly focused on large language models, leading to the development of powerful models such as GPT-3 [12], GPT-4 [13], LlaMA [14], Mixtra [15], and others. The GPT (Generative Pre-trained Transformer) is a deep learning model based on the transformer architecture [16], specifically designed for natural language processing (NLP) tasks. GPT processes text sequences using a stack of self-attention layers, each comprising multiple attention heads and feedforward neural networks. The model employs a unidirectional autoregressive approach to predict the next token in a sequence based on the preceding tokens. Pre-training involves the prediction of tokens in large datasets to capture linguistic patterns and contextual meanings.

Generative language models in chemistry, also known as Chemical language models (CLMs), leverage deep learning to process vast datasets of chemical compounds, biological interactions, and molecular properties. These models enable researchers to design new molecules that target specific proteins or pathways [17].

Several current initiatives utilize LLMs for molecular design and drug discovery, including GPT-based models, like MolGPT. MolGPT uses scaffold-based Simplified Molecular-Input Line-Entry System (SMILES) strings paired with target properties to design the desired molecule. Research shows that MolGPT is highly effective at generating molecules with specific target properties, surpassing the performance of traditional approaches and other deep-learning models [18]. A more promising research direction involves the development of generative models capable of producing valid SMILES representations conditioned on specific target sequences [19]. Furthermore, given the extensive and continuously expanding body of scientific literature, it is imperative that such systems incorporate mechanisms for the autonomous identification and prioritization of relevant disease-associated targets.

Recent AI development focuses on creating AI agents that are utilized to automate complex tasks, enhance decision-making, and process vast datasets with high efficiency and accuracy [20]. Given the complexity and vast scale of biological and chemical data, the goal of our work is to develop an integrated AI system that employs three deep learning models to identify drug targets, generate molecules depending on the amino acid sequence of the target, and calculate DTIs.

## 2. Materials & Methods

### 2.1. AgentMol System Overview

**Figure 1:**
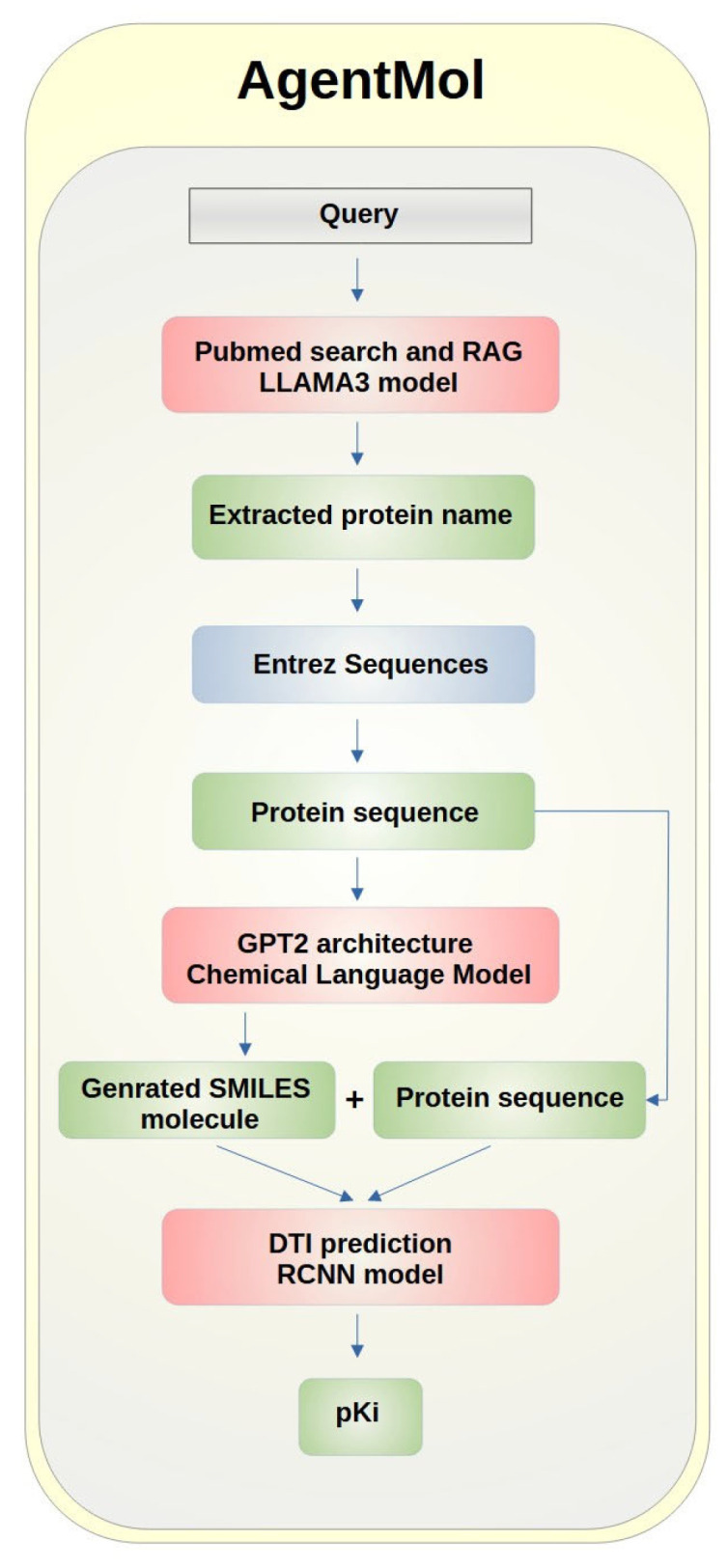
AgentMol pipeline. RAG—Retrieval-Augmented Generation, GPT-2—Generative Pre-trained Transformer 2, DTI—drug target interactions, RCNN—Regression convolutional neural network, pKi—−log inhibition constant.

AgentMol (Figure 1) is an integrated computational system designed to facilitate drug discovery using advanced artificial intelligence models. The process initiates with a user query, which is interpreted by the LLaMA3 language model in conjunction with a PubMed-based RAG mechanism to identify and extract the target protein name. Subsequently, the corresponding first protein sequence is retrieved from the Entrez database.

A generative chemical model, architecturally based on GPT-2, is then employed to translate the protein sequence into a candidate small molecule represented in SMILES format. To assess the interaction potential between the generated molecule and the target protein, a regression convolutional neural network (RCNN) model is utilized. This model integrates both the SMILES representation and the protein sequence to predict the pKi value, a quantitative measure of binding affinity. All components of AgentMol are orchestrated using the LangGraph framework [21].

### 2.2. Query and LLaMA3 Model

In our study, we implemented a retrieval-augmented pipeline to extract relevant biomedical literature based on user-defined input queries. The process leverages an open-source large language model LLaMA3 and a specialized retriever module tailored for scientific document retrieval. Specifically, we utilized the OllamaLLM class, an abstraction for interfacing with a pre-trained language model, initialized with a configurable model parameter stored in the “state[“option”]” variable. To retrieve protein names, we employed the PubMedRetriever class, a retrieval tool designed to query the PubMed database, which contains a vast repository of peer-reviewed biomedical and life sciences literature. The following sequence is added to each prompt: “extract all best matches protein name abbreviations from a given text and list them using * (your output should only be this list without any other words and sentences)”.

### 2.3. GPT2 Based Chemical Language Model

GPT-2 model architecture consists of a stack of identical transformer decoder layers, with each layer containing multi-head self-attention and position-wise feedforward neural networks. It uses layer normalization and masked self-attention to ensure that each token only attends to previous tokens. The model relies on positional embeddings to handle sequential data input and can be pre-trained on large corpora using unsupervised learning with a language modeling objective predicting the next token in a sequence [22].

The GPT-2–based chemical language model was trained using paired ligand–protein sequences contained in CSV files, where SMILES and amino acid sequences were merged into a single input column. A Byte-Pair Encoding (BPE) tokenizer was trained from scratch on the training corpus using the Hugging Face tokenizers library, with a vocabulary size of 30,000 and a minimum token frequency threshold of 2. The tokenizer incorporated special tokens (<bos>, <eos>, <pad>, <unk>) and used whitespace pre-tokenization to preserve sequence-level structure. Each input sequence was truncated or padded to a maximum length of 128 tokens. Model training was performed with a GPT-2 configuration comprising a context window of 1024 tokens and initialized using random weights. Training was conducted for 10 epochs with a batch size of 64, learning rate of 5 × 10^−4^, cosine learning rate scheduling, 1000 warmup steps, and weight decay of 0.1. Gradient accumulation was set to 8 to stabilize optimization on limited computational resources. Model performance was periodically evaluated every 5000 steps, with training and validation losses logged via a custom callback function. The Data Collator For Language Modeling utility was used to dynamically mask sequences for autoregressive generation during training.

Models were trained from scratch using the Python library Transformers (version 4.46.0) [23]. The data were split into a training set (70%) and a validation set (30%) based on ligand-protein sequence pairs. A test set consisting of one thousand amino acid sequences was reserved from the original dataset and used with the trained model to generate sequences. Raw textual input was converted into tokenized representations, which were numerical encodings processed by the model. The primary training parameters are presented in Table 1.

The trained models generated the SMILE sequence based on 1000 amino acid protein sequences of the test set. Generation parameters were: max new tokens equal 100, temperature equal 1, early stopping set on True.

We have observed over-generation of sequences, which resulted in a low percentage of validity (21%), therefore SMILES sequences have been automatically trimmed to valid sequences. The trimming algorithm iteratively shortens each generated SMILES string from the end by removing one character at a time until a syntactically valid molecule is obtained or the string becomes empty. For each SMILES sequence, validity is assessed using the RDKit Chem.MolFromSmilesfunction.

### 2.4. Regression Convolutional Neural Network Model for DTI Prediction

**Figure 2:**
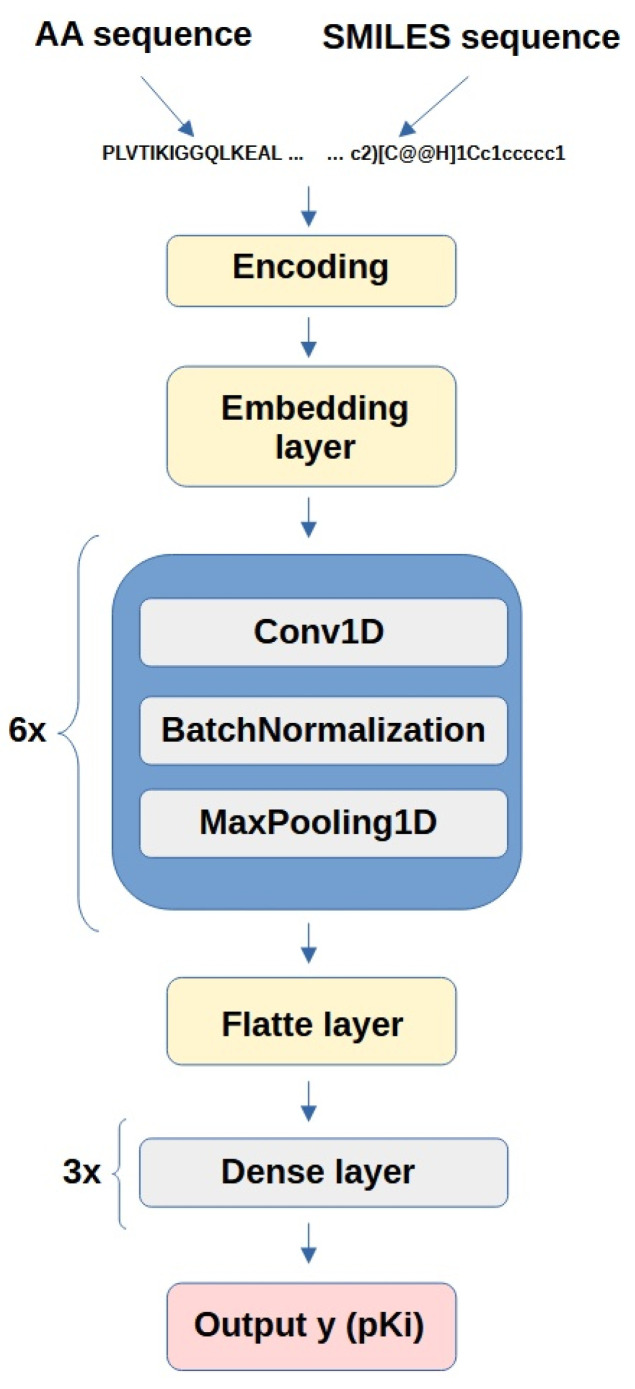
Architecture of the Regression Convolutional Neural Network model for DTI prediction.

Similarly to the GPT-2 model, the dataset was divided into a training set and validation set at a 70/30 ratio. For the input representation, we assigned numbers to words, where each sequential character corresponded to an integer ranging from zero to 70. These feature vectors were fed into the neural network models. The model architecture is shown in Figure 2.

The input sequences, consisting of concatenated amino acid and SMILES strings, were to kenized at the character level using the Keras Tokenizer and padded or truncated to 800 tokens. The embedding layer converted integer-encoded tokens into 8-dimensional dense vectors. The model architecture comprised six one-dimensional convolutional layers with kernel sizes of 8, filter numbers from 32 to 64, and batch normalization, each followed by max-pooling (size = 2). Leaky ReLU activation (α = 0.01) was applied to all convolutional layers. The extracted features were flattened and passed through two fully connected layers (512 and 10 neurons) and a final linear output predicting continuous pKi values.

Training was performed using RMSprop (learning rate = 0.001) for 200 epochs. Mean absolute error (MAE) and mean squared error (MSE) served as loss functions, while Pearson’s correlation coefficient (R), coefficient of determination (R^2^), and root mean squared error (RMSE) were used for evaluation. Each experiment was repeated five times with randomized data partitions to assess model stability. All computations were carried out using TensorFlow v2.9.1 and Keras v2.9.0. [24].

### 2.5. Dataset and Data Preprocessing

Models were trained and evaluated using BindingDB database (http://www.bindingdb.org) [25]. The SMILES of the small-molecule compounds was combined with the amino acid sequence of the protein targets into a pair of ligand-protein sequences (n = 470,560). Sequences with more than 800 characters were excluded from the analysis. The average length of the sequence pairs was 472.56 with a minimum and maximum value of 35 and 799, respectively (Figure S1, Table 2).

For model training values of Ki (nM) was transformed into log space, pKi [8]. Values greater than 10 were excluded.

After transformation the mean pKi value was 6.79 and minimal value was 0.002 (Figure S2, Table 2).

## 3. Results

### 3.1. Chemical Language Model Performance

Table 3 summarizes the performance of the GPT-2-based chemical language model across key evaluation metrics. Validity refers to the proportion of syntactically valid chemical structures generated, with a perfect score of 1.00 indicating all generated molecules are valid. Diversity (0.89) quantifies the structural variety among generated molecules, while uniqueness (0.96) measures the fraction of non-redundant outputs. QED (Quantitative Estimate of Drug-likeness) is standard cheminformatics metric to assess the drug-like quality of molecules, with a mean value of 0.50 indicating moderate drug-likeness among generated compounds.

### 3.2. RCNN Performance Results

The loss function was monitored during training. In the RCNN model, the validation set loss function remained stable at approximately epoch 50, with a constant decline in the training set (Figure S3).

The loss function after 200 epochs on the validation set for the RCNN model was 0.907 for the MSE and 0.656 for the MAE training. For MSE and MAE, Pearson’s correlation coefficient R was greater than 0.8, RMSE > 0.9, and R^2^ more than 0.6 (Table 4).

The prediction of pKi on the 1000 CLM generated SMILES sequences and their corresponding amino acid sequences was 6.49 and was similar to the overall average pKi of the training set (6.79).

### 3.3. User Case

In a representative use case (Figure S4), the user provided the prompt “lung cancer protein biomarker” to AgentMol. The system first processed the query using the LLaMA3 language model in conjunction with a PubMed-based RAG mechanism, which identified and extracted the target protein SP100. The corresponding protein sequence was then retrieved from the Entrez database. Using this sequence as input, the GPT-2-based generative chemical model produced a candidate small molecule represented in SMILES format as Nc1ncnc2n(cnc12)[C@@H]. To evaluate the potential interaction between the generated molecule and SP100, a regression convolutional neural network model was applied, integrating both the SMILES representation and the protein sequence to predict binding affinity. The model estimated a pKi value of 5.08, indicating moderate binding affinity. This example illustrates the end-to-end workflow of AgentMol, from a user query to target identification, molecule generation, and computational affinity prediction.

## 4. Discussion

The development of AgentMol represents a step forward in the integration of multi-model AI systems for automated drug discovery. The combination of retrieval-augmented generation, chemical language modeling via GPT-2 architecture, and drug–target interaction prediction using a regression convolutional neural network creates a coherent and end-to-end system capable of identifying drug targets, generating novel molecules, and evaluating binding affinity.

Models were selected for their open-access nature and low computational requirements.

The use of LLaMA3 in a RAG-based search scenario demonstrates its ability to extract relevant protein targets from complex biomedical texts, significantly enhancing the early phase of drug discovery.

The GPT-2-based chemical language model demonstrated high syntactic validity (1.00) and uniqueness (0.96), with notable diversity (0.89) in generated compounds similar to target-aware generative models (Table 5).

The moderate mean QED score (0.5) likely reflects the model’s primary training objective, which emphasized learning chemical syntax and protein–ligand relationships rather than explicitly optimizing for pharmacokinetic or physicochemical properties. Since QED is influenced by factors such as molecular weight, lipophilicity, and specific functional group composition—none of which were directly constrained during training—the resulting chemical space encompasses both highly and moderately drug-like molecules. Such diversity may be advantageous during the early exploration phase of drug discovery, as it promotes chemical novelty. Nonetheless, future work could incorporate QED-based or multi-objective optimization frameworks to bias generation toward candidates with enhanced pharmacological relevance and improved drug-like characteristics. The model was trained from scratch, which affirms the feasibility of applying generalized NLP architectures directly to chemical sequence generation with domain-specific data. obtaining similar validation values, in contrast to other chemical GPT models [18,27,28], our model was trained together with the amino acid sequence of the protein. AgentMol model is trained using paired ligand-protein sequences, effectively enabling protein-conditioned molecular generation. This introduces biological context directly into the generative process, offering the potential to tailor molecules more precisely toward target-specific interactions. Such conditioning could lead to increased hit rates in early screening stages and may reduce the need for exhaustive downstream filtering. By conditioning generation on protein sequences, the model may learn latent representations that reflect the physicochemical or structural preferences of specific targets. Although the model operates in 1D sequence space, this architecture opens the door for interpretable attention patterns between amino acid motifs and generated substructures, however 1D sequence space may limit the biological plausibility of generated molecules, particularly for targets requiring specific 3D binding conformations. However, determining protein tertiary structures is often expensive and experimentally challenging, and many proteins lack sufficient structural data or known ligands. In contrast, the presented approach relies exclusively on primary amino acid sequences, enabling molecule generation without predefined chemical descriptors or 3D information. This sequence-based framework thus offers a more accessible and generalizable strategy for early-stage drug discovery, particularly when structural data are incomplete or unavailable. Future improvements could focus on integrating 3D molecular structure information to enhance the biological realism and precision of ligand–target interactions. Incorporating structural descriptors or embeddings derived from protein conformations could improve the model’s ability to capture spatial complementarity. Additionally, evaluating the model’s generalizability across unseen or evolutionarily distant protein families would provide a more comprehensive assessment of its robustness and practical applicability.

The comparison of DTI regression models is presented in Table 6.

Although the RCNN model achieved satisfactory performance metrics (R > 0.8, RMSE > 0.9, R^2^ > 0.6), the R^2^ value indicates that a proportion of variance in experimental pKi values remains unexplained. This may be attributed to the inherent complexity and noise of biological binding data, as well as the limited representation of certain affinity ranges within the training set. To assess potential overfitting, training and validation losses were monitored throughout the optimization process, showing stable convergence after approximately 50 epochs.

The integration of LangGraph into the AgentMol system provides a robust framework for orchestrating a multi-model AI pipeline by structuring model components as modular, interoperable nodes. This architecture enables efficient execution, state tracking, and fault-tolerant handling of complex tasks such as literature retrieval, molecule generation, and affinity prediction. As a graph-based agent system, LangGraph enhances scalability and maintainability, making it well-suited for adaptive and iterative workflows in computational drug discovery. LangGraph’s modular design facilitates extensibility, allowing individual components to be independently updated, replaced, or expanded without altering the overall system architecture [21].

The conceptual novelty of AgentMol lies in the direct integration of retrieval-augmented generation (RAG), sequence-based molecular generation, and affinity prediction within a single, modular framework. In contrast to prior generative AI systems that address molecule design and target prediction as independent stages, AgentMol establishes a unified workflow that begins with automatic identification of potential protein targets directly from biomedical abstracts using the RAG module. This retrieved contextual information guides the subsequent GPT-2–based molecular generation conditioned on protein sequences, effectively linking target discovery with compound design. The generated molecules are then evaluated by the RCNN model for their predicted binding affinity, forming an end-to-end, knowledge-driven system that seamlessly connects information retrieval, molecular generation, and predictive evaluation within a single automated pipeline.

Streamlit (v1.45.0) was used to develop the AgentMol web application due to its simplicity, rapid prototyping capabilities, and seamless integration with Python-based machine learning workflows. Its interactive interface allows users to input queries, visualize results, and run complex models in real time without requiring extensive frontend development.

Our system combines, for the first time, a disease target finding model that generates a molecule and its affinity for the target. The proposed models do not require much computing power and can be trained and used on the CPU.

Recent advances in drug–drug interaction (DDI) prediction further illustrate the growing impact of large language models in molecular representation learning. For instance, the LLM-DDI framework combines GPT-based molecular embeddings with graph neural networks to capture semantic relationships within biomedical knowledge graphs, achieving state-of-the-art performance on real-world datasets [30].

A notable limitation of the present study lies in the potential bias inherited from the BindingDB dataset. Although BindingDB provides a large and diverse collection of ligand–protein interactions, its distribution across protein families and ligand chemotypes is inherently unbalanced. Such uneven representation may lead the model to favor well-represented target classes while underperforming on rare or poorly characterized proteins. This limitation could partially explain performance variability across different targets and highlights the importance of dataset diversity in model generalization. Future work will address this issue by incorporating data from complementary sources such as ChEMBL or PDBbind and by applying sequence-based clustering to ensure more balanced target representation. In addition, evaluating stratified performance metrics by protein family, functional class, or sequence length could provide deeper insights into model robustness and help identify systematic biases affecting underrepresented targets. Future investigation will also focus on integrating multi-objective optimization, structural validation, and feedback from downstream bioactivity predictions to improve candidate quality and relevance. To validate the clinical relevance of AgentMol’s predictions, subsequent studies should incorporate experimental validation through wet-lab binding assays, such as surface plasmon resonance or isothermal titration calorimetry, to confirm the predicted binding affinities of generated molecules against their target proteins. Cross-validation with orthogonal computational methods, such as molecular docking or molecular dynamics simulations, would further strengthen confidence in the biological plausibility of generated candidates. Such a multi-tiered validation strategy would bridge the gap between computational predictions and experimental druggability, ultimately accelerating the translation of AI-generated molecules into viable therapeutic leads.

These findings highlight the potential of combining general-purpose language models with domain-specific training and predictive modules to accelerate early-stage drug discovery in a resource-efficient and reproducible manner.

## Figures and Tables

**Table 1:** GPT-2 model training arguments.

Value	Argument
steps	evaluation_strategy
5000	eval_steps
8	gradient_accumulation_steps
10	num_train_epochs
0.1	weight_decay
1000	warmup_steps
cosine	lr_scheduler_type
5.00 × 10^−4^	learning_rate

**Table 2:** Descriptive statistics of models input dataset after preprocessing.

	Length	pKi
n	470,560	470,560
mean	472.56	6.79
std	123.23	1.49
min	35.0	0.002
25%	400.0	5.74
50%	479.0	6.87
75%	531.0	7.92
max	799.0	9.99

**Table 3:** Evaluation metrics for the GPT-2 chemical language model.

Parameter	Value
Validity	1
Diversity	0.89
Unique	0.96
QED	0.5

**Table 4:** Performance of RCNN model.

Loss Function and Set	Pearson’s Correlation R—Mean (SD)	RMSE 200 Epoch—Mean (SD)	RMSE 1 Epoch—Mean (SD)	R2 200 Epoch—Mean (SD)	R2 After 1 Epoch—Mean (SD)	Loss After 200 Epoch—Mean (SD)	Loss After 1 Epoch—Mean (SD)
MSE training set	0.977 (0.002)	0.359 (0.005)	1.388 (0.006)	0.947 (0.001)	0.214 (0.007)	0.129 (0.003)	1.927 (0.017)
MSE validation set	0.805 (0.002)	0.952 (0.012)	1.358 (0.108)	0.628 (0.008)	0.241 (0.120)	0.907 (0.024)	1.854 (0.290)
MAE training set	0.955 (0.020)	0.408 (0.013)	1.433 (0.018)	0.931 (0.004)	0.158 (0.021)	0.260 (0.008)	1.129 (0.016)
MAE validation set	0.808 (0.004)	0.933 (0.019)	1.342 (0.062)	0.645 (0.015)	0.264 (0.070)	0.656 (0.018)	1.061 (0.056)

**Table 5:** Comparison of target-aware generative models in terms of QED, validity, novelty, and uniqueness.

Model	QED	Validity	Novelty	Uniqueness
AgentMol	0.5	1	0.89	0.96
DeepTarget [19]	0.66	0.8083	0.99	0.99
DeepDTAGen (SMILES) [26]	0.59	0.99	0.99	0.22

**Table 6:** Comaprison of R^2^ of various predictive regression models.

Model	R^2^
AgentMol	0.645 (0.015)
SimBoost [29]	0.644 (0.006)
DeepDTA [8]	0.636 (0.006)

## Data Availability

Trainig and evaluation datasets, model training scripts, models, generated results and streamlite web aplication are made available on the Zenodo platform which can be retrieved using the following link https://zenodo.org/records/15296657 (accessed on 23 November 2025).

## Supplementary Materials

The following supporting information can be downloaded at: https://www.mdpi.com/article/10.3390/mps8060143/s1, Figure S1: Distribution of ligand–protein sequence lengths in the BindingDB training dataset; Figure S2: Density of logarithmic Ki values; Figure S3: Example of the training and validation loss curves plot for a convolutional neural network RCNN model over 200 epochs; Figure S4: Representative interface view of the AgentMol application demonstrating a user case scenario.

## Abbreviations

The following abbreviations are used in this manuscript:

AI	Artificial Intelligence
GPT	Generative pre-trained transformer
DTI	Drug target interactions
LLM	Large language model
Ki	Inhibition constant
Kd	Dissociation constant
CNN	Convolutional neural network
NLP	Natural language processing
CLM	Chemical language model
SMILES	Simplified Molecular-Input Line-Entry System
RAG	Retrieval-Augmented Generation
RCNN	Regression convolutional neural network
pKi	−log inhibition constant
MAE	Mean absolute error
MSE	Mean squared error
RMSE	Root mean squared error
QED	Quantitative Estimate of Drug-likeness

## References

[1] Kumar M., Nguyen T.P.N., Kaur J., Singh T.G., Soni D., Singh R., Kumar P. (2023). Opportunities and challenges in application of artificial intelligence in pharmacology. Pharmacol. Rep..

[2] Wan F., Zhu Y., Hu H., Dai A., Cai X., Chen L., Gong H., Xia T., Yang D., Wang M.-W. (2019). DeepCPI: A Deep Learning-Based Framework for Large-Scale in Silico Drug Screening. Genom. Proteom. Bioinform..

[3] Chen W., Liu X., Zhang S., Chen S. (2023). Artificial intelligence for drug discovery: Resources, methods, and applications. Mol. Ther. Nucleic Acids.

[4] Chakraborty C., Bhattacharya M., Lee S.-S. (2023). Artificial intelligence enabled ChatGPT and large language models in drug target discovery, drug discovery, and development. Mol. Ther. Nucleic Acids.

[5] Rifaioglu A.S., Atalay R.C., Kahraman D.C., Doğan T., Martin M., Atalay V. (2020). MDeePred: Novel multi-channel protein featurization for deep learning-based binding affinity prediction in drug discovery. Bioinformatics.

[6] Zhao W., Yu Y., Liu G., Liang Y., Xu D., Feng X., Guan R. (2024). MSI-DTI: Predicting drug-target interaction based on multi-source information and multi-head self-attention. Briefings Bioinform..

[7] Thafar M.A., Alshahrani M., Albaradei S., Gojobori T., Essack M., Gao X. (2022). Affinity2Vec: Drug-target binding affinity prediction through representation learning, graph mining, and machine learning. Sci. Rep..

[8] Öztürk H., Özgür A., Ozkirimli E. (2018). DeepDTA: Deep drug–target binding affinity prediction. Bioinformatics.

[9] Lee I., Keum J., Nam H. (2019). DeepConv-DTI: Prediction of drug-target interactions via deep learning with convolution on protein sequences. PLOS Comput. Biol..

[10] Rezaei M.A., Li Y., Wu D., Li X., Li C. (2022). Deep Learning in Drug Design: Protein-Ligand Binding Affinity Prediction. IEEE/ACM Trans. Comput. Biol. Bioinform..

[11] Wang K., Zhou R., Li Y., Li M. (2021). DeepDTAF: A deep learning method to predict protein–ligand binding affinity. Briefings Bioinform..

[12] Brown T.B., Mann B., Ryder N., Subbiah M., Kaplan J., Dhariwal P., Neelakantan A., Shyam P., Sastry G., Askell A. (2020). Language Models Are Few-Shot Learners. arXiv.

[13] Achiam J., Adler S., Agarwal S., Ahmad L., Akkaya I., Aleman F.L., Almeida D., Altenschmidt J., Alt-man S., OpenAI (2023). GPT-4 Technical Report. arXiv.

[14] Touvron H., Lavril T., Izacard G., Martinet X., Lachaux M.-A., Lacroix T., Rozière B., Goyal N., Hambro E., Azhar F. (2023). LLaMA: Open and Efficient Foundation Language Models. arXiv.

[15] Jiang A.Q., Sablayrolles A., Roux A., Mensch A., Savary B., Bamford C., Chaplot D.S., de las Casas D., Hanna E.B., Bressand F. (2024). Mixtral of Experts. arXiv.

[16] Vaswani A., Shazeer N., Parmar N., Uszkoreit J., Jones L., Gomez A.N., Kaiser L., Polosukhin I. (2017). Attention Is All You Need. arXiv.

[17] Grisoni F. (2023). Chemical language models for de novo drug design: Challenges and opportunities. Curr. Opin. Struct. Biol..

[18] Bagal V., Aggarwal R., Vinod P.K., Priyakumar U.D. (2021). MolGPT: Molecular Generation Using a Transformer-Decoder Model. J. Chem. Inf. Model..

[19] Chen Y., Wang Z., Wang L., Wang J., Li P., Cao D., Zeng X., Ye X., Sakurai T. (2023). Deep generative model for drug design from protein target sequence. J. Cheminformatics.

[20] Renney H., Nethercott M., Williams O., Evetts J., Lang J. Reimagining the Data Landscape: A Multi-Agent Paradigm for Data Interfacing. Proceedings of the 2025 8th International Conference on Data Science and Machine Learning Applications (CDMA).

[21] Wang J., Duan Z. (2024). Agent AI with LangGraph: A Modular Framework for Enhancing Machine Translation Using Large Language Models. arXiv.

[22] Radford A., Wu J., Child R., Luan D., Amodei D., Sutskever I. Language Models Are Unsupervised Multitask Learners; OpenAI Blog 2019. https://cdn.openai.com/better-language-models/language_models_are_unsupervised_multitask_learners.pdf.

[23] Wolf T., Debut L., Sanh V., Chaumond J., Delangue C., Moi A., Cistac P., Rault T., Louf R., Funtowicz M. (2020). HuggingFace’s Transformers: State-of-the-Art Natural Language Processing. arXiv.

[24] Abadi M., Agarwal A., Barham P., Brevdo E., Chen Z., Citro C., Corrado G.S., Davis A., Dean J., Devin M. (2016). Ten-sorFlow: Large-Scale Machine Learning on Heterogeneous Distributed Systems. arXiv.

[25] Liu T., Lin Y., Wen X., Jorissen R.N., Gilson M.K. (2007). BindingDB: A web-accessible database of experimentally determined protein-ligand binding affinities. Nucleic Acids Res..

[26] Shah P.M., Zhu H., Lu Z., Wang K., Tang J., Li M. (2025). DeepDTAGen: A multitask deep learning framework for drug-target affinity prediction and target-aware drugs generation. Nat. Commun..

[27] Ye G. (2024). De novo drug design as GPT language modeling: Large chemistry models with supervised and reinforcement learning. J. Comput. Mol. Des..

[28] Thomas M., O’bOyle N.M., Bender A., De Graaf C. (2024). MolScore: A scoring, evaluation and benchmarking framework for generative models in de novo drug design. J. Cheminformatics.

[29] He T., Heidemeyer M., Ban F., Cherkasov A., Ester M. (2017). SimBoost: A read-across approach for predicting drug–target binding affinities using gradient boosting machines. J. Cheminformatics.

[30] Li D., Yang Y., Cui Z., Yin H., Hu P., Hu L. (2025). LLM-DDI: Leveraging Large Language Models for Drug-Drug Interaction Prediction on Biomedical Knowledge Graph. IEEE J. Biomed. Heal. Informatics.

