# Supplementary material for: AgentMol: Multi-Model AI System for Automatic Drug-Target Identification and Molecule Development
Source: Methods Protoc. 2025 Dec 1;8(6):143. doi: 10.3390/mps8060143 (PMC12736193; doi:10.3390/mps8060143)
Supplement: Supplementary file 1 [file mps-08-00143-s001.zip › mps-3943570-supplementary.pdf]

Supplementary Materials

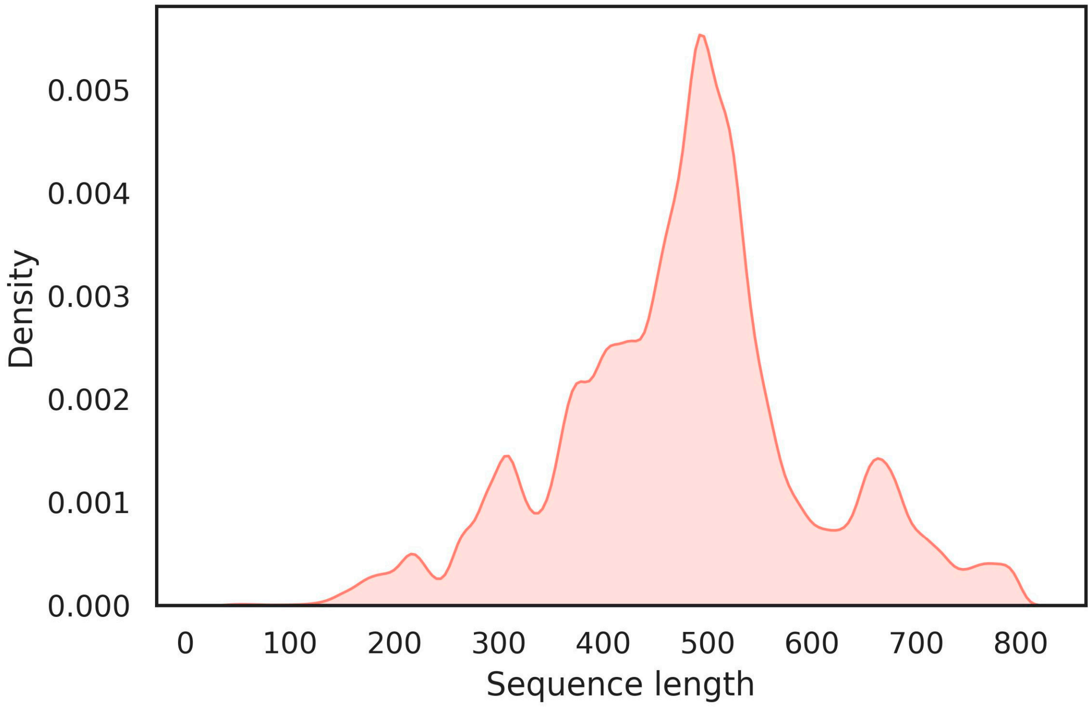

Figure S1. Distribution of ligand–protein sequence lengths in the BindingDB training dataset.

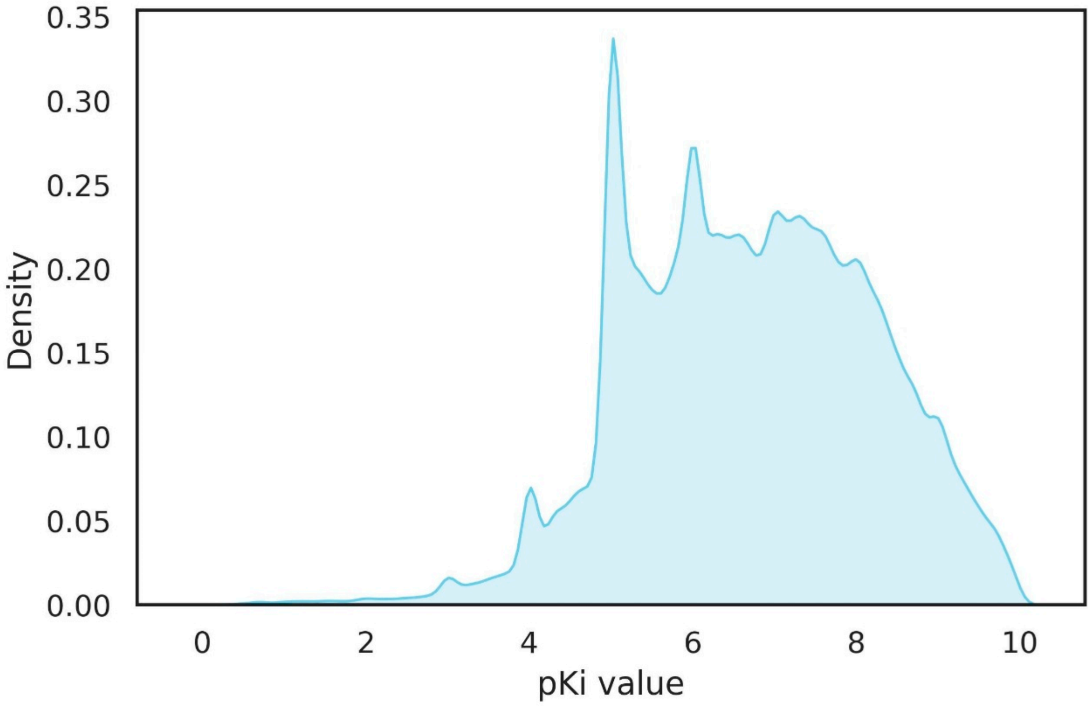

Figure S2. Density of logarithmic Ki values.

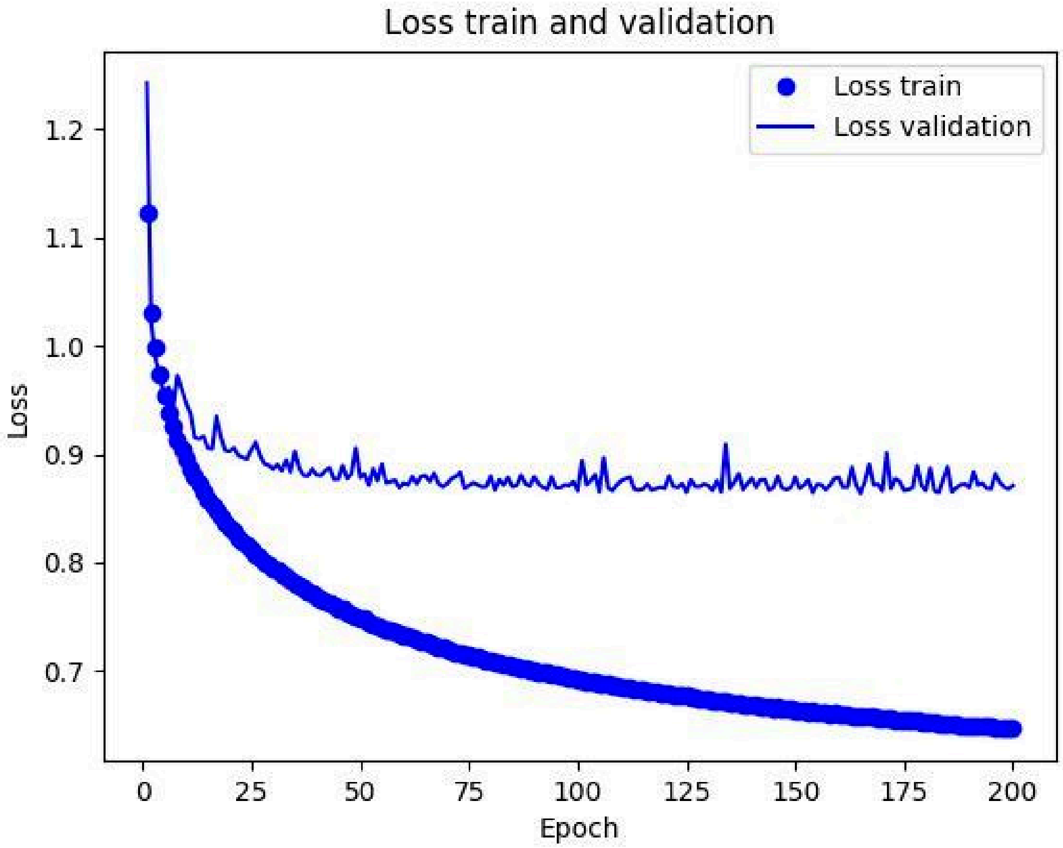

Figure S3. Example of the training and validation loss curves plot for a convolutional neural network RCNN model over 200 epochs.

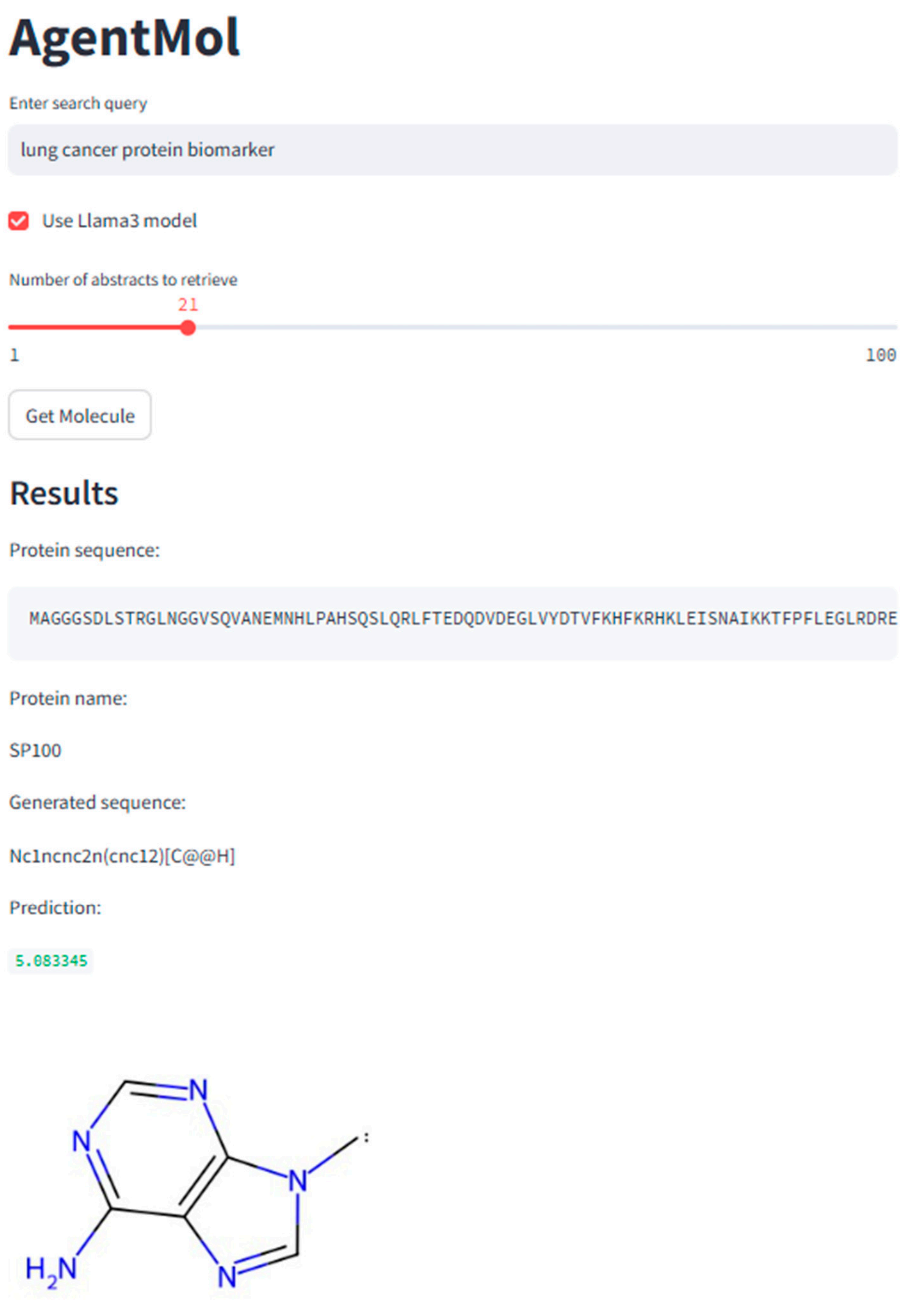

Figure S4. Representative interface view of the AgentMol application demonstrating a user case scenario.
